# Reliability and validity of the online Pittsburgh sleep quality index in college students from low-income regions

**DOI:** 10.3389/fdgth.2024.1394901

**Published:** 2024-07-24

**Authors:** Augusto César Ferreira De Moraes, Lorrane Cristine Conceição da Silva, Barbara Saldanha Lima, Kliver Antonio Marin, Ethan T. Hunt, Marcus Vinicius Nascimento-Ferreira

**Affiliations:** ^1^ The University of Texas Health Science Center at Houston, School of Public Health in Austin, Department of Epidemiology, Michael & Susan Dell Center for Healthy Living, Texas PARC - the Texas Physical Activity Research Collaborative Lab, Austin, TX, United States; ^2^ Graduate Program in Public Health, Graduate Program in Epidemiology, School of Public Health, University of Sao Paulo, Sao Paulo, Brazil; ^3^ YCARE (Youth/Child and CArdiovascular Risk and Environmental) Research Group, Faculdade de Medicina, Universidade de Sao Paulo, São Paulo, Brazil; ^4^ Health, Physical Activity and Behavior Research (HEALTHY-BRA) Group, Universidade Federal do Tocantins, Miracema do Tocantins, Brazil; ^5^ Instituto de Ensino Superior do Sul do Maranhão (IESMA/UNISULMA), Imperatriz, Brazil; ^6^ The University of Texas Health Science Center at Houston, School of Public Health in Austin, Department of Health Promotion and Behavioral Science, Michael & Susan Dell Center for Healthy Living, Texas PARC - the Texas Physical Activity Research Collaborative Lab, Austin, TX, United States

**Keywords:** sleep quality, sleep duration, psychometric properties, surveys and questionnaires, college students

## Abstract

**Objectives:**

We aimed to test the reliability and structural validity (also called dimensionality) of the online Pittsburgh Sleep Quality Index among college students from low-income regions.

**Methods:**

We assessed 195 Brazilian college students from a low-income region (Gini index of 0.56), of whom 117 were reassessed to evaluate the reliability. We collected all data in a self-reported online twice, 2-week apart. We evaluated reliability and structural validity.

**Results:**

All questionnaire components showed reliability, correlation coefficient ≥0.49. In the structural validity, the confirmatory analysis showed better global model adjustment for the one-factor (RMSEA = 0.019; SRMR = 0.041; CFI = 0.992; TLI = 0.986) solution compared with two-factor (RMSEA = 0.099; SRMR = 0.070; CFI = 0.764; TLI = 0.619) and three-factor (RMSEA = 0.108; SRMR = 0.066; CFI = 0.763; TLI = 0.548) solutions, respectively.

**Discussion:**

The online questionnaire presents acceptable reliability and structural validity in Brazilian low-income regions.

## 1 Introduction

Sleep is crucial for optimal health. However, irregular work schedules, shift patterns, and poor sleep habits often lead to abnormal sleep quantity and quality in adults (1). In academia, the high prevalence of poor sleep quality among college students in low- and lower-middle-income countries represents a significant public health concern (2). Factors such as stress, insomnia, being in the second year, bedtime electronic device usage, and chronic illnesses are key determinants of sleep quality in this group (2). Monitoring sleep quality has the potential to enhance adult health, especially within the academic context.

Subjective measurement tools can be used in clinical practice for diagnostic purposes, for monitoring treatment responses, or in epidemiological studies to measure the quality of sleep on population level (3). These tools have the advantages of cost effectiveness, ease of administration and high patient compliance. In this context, scientists validated the Brazilian version of Pittsburgh Sleep Quality Index (PSQI-BR) questionnaire measured in person against overnight polysomnography (PSG) and concluded PSQI-BR is a valid and reliable instrument for the assessment of sleep quality in Brazilian populations (4). However, we emphasize that the evaluation was carried out with interviews in a clinical context, and when applying the same questionnaire in free-living population by self-report or in online formats, these results may be different (5). In addition, the PSQI dimensionality is much debated. A comprehensive systematic literature review indicated that one- to three-factor were the possible dimensionality for this subjective tool (6). In this regard, there is no consensus about the PSQI dimensionality in the literature. Mainly because the factor structure is easily influenced by sampled data (e.g., across different groups or over time); and, repetitive revalidation studies are needed to overcome the sampling bias and to confirm the latent variable structure (7). A psychometric evaluation comparing different dimensionalities (two- vs. three-model structure) in different timepoints identified two-factor as appropriated solution in Singaporean adults (8). Although there is no dimensionality assessment of the PSQI-BR in Brazilian adults, evidences in adolescents also indicated two-factor structure solution (9).

The surge in COVID-19-related research has witnessed the migration of numerous tools to online platforms (10). However, the literature is scarce on the psychometric properties of the online format of the PSQI, although the level of education of the participants and access to the internet may distort recruitment and the ability to respond to online tools, mainly in populations with marked socioeconomic and educational differences (10), which is the Brazilian case. Thus, when one wishes to apply these tools in other populations, proper cross-cultural adaptation, questionnaire administration and validation are required (11). Therefore, we hypothesized that i) the online PSQI-BR has acceptable reliability and structural validity in Brazilian low-income regions, as well as, ii) the scale presents dimensionality based on two-factor solution with a new dataset of college students.

## 2 Material and methods

### 2.1 Study design

The current project consists of a methodological study (11) belonging to the first stage of a multicenter longitudinal observational project entitled: 24-hour movement behavior and metabolic syndrome (24h-MESYN), a prospective cohort study on lifestyle and risk of developing metabolic syndrome in undergraduate students from low-income regions during a pandemic (12). In this phase of the study, the participants answered the same questionnaire twice, 2 weeks apart, in order to verify the reliability (temporal stability of the responses) and the construct (structural) validity (12). Data collection took place during the first half of 2021.

### 2.2 Ethical aspects

The project was approved by the Research Ethics Committee of *Universidade Ceuma* (ID: 4,055,604) and *Universidade Federal do Tocantins* (ID: 5,161,340), respectively. The project follows the ethical principles for research with human beings of the Declaration of Helsinki, revised in 2008, Seoul, Korea; the resolution of CNS 466/12; guidelines for conducting research activities during the pandemic caused by COVID-19 (available at: www.fo.usp.br/wp-content/uploads/2020/07/Orientações-conduza-de-pesquisa-etividades-CEP.pdf) and guidelines for research in a virtual environment (OFÍCIO CIRCULAR NO. 2/2021/CONEP/SECNS/MS). After the institution's written consent, the selected students received a formal and detailed invitation to participate about the study objectives, risks and benefits, so they had the opportunity to clarify any doubts and could voluntarily consent to collaborate with the research. In case of interest in participating in the study, the student was invited to sign an informed consent form (online).

### 2.3 Population and sample size

This project consisted of students enrolled in a higher education institution in the region of the state of Maranhão, Brazil, selected for convenience. The city has a Gini index of 0.56 (13). In 2020, the institution had 2,225 students enrolled in 9 undergraduate courses (Administration, Law, Physical Education, Nursing, Aesthetics and Cosmetics, Physiotherapy, Nutrition, Psychology and Social Work). The sample was calculated according to Nascimento-Ferreira's assumptions (14). The parameters used to calculate the sample size were: α of 0.05, β of 0.10 (or power of 90%), number of replicates of 2 and intraclass correlation coefficient of 0.65 (9). From these parameters, we estimated a sample of 41 individuals. Predicting 50% losses and rejections, in the first and second application, we established a minimum sample size of 62 participants to study the psychometric properties of the Pittsburgh Sleep Quality Index. However, the 24 h-MESYN project was designed to evaluate the psychometric properties of several epidemiological tools. Based on estimates of less robust psychometric properties, we invited 342 participants aiming to fulfill minimum aspects (α of 0.05 and β of 0.10) for all tested tools (12). The sample diversity was based on a stratified random distribution of students, according to sex (at least 60.0% for female biological sex), age (at least 25.0% for students up to 20 years old) and study program (at least 60.0% ongoing in the health area), based on previous representative cohort study (15).

### 2.4 Inclusion and exclusion criteria

All students had to enrolled at the Institution regularly, be at least 17 years of age, and provide signed an informed consent form were included in the study. Students aged 17 provided assent agreements, along with consent from their parent or legal guardian. The study exclusion criteria were: pregnancy, physical disability or failure to complete the questionnaires. In the case of pregnancy and physical disability, the students were evaluated, but excluded from the analyzes.

### 2.5 Study variables and instruments

The study variables were biological sex, age, nature of the course, academic period and sleep disorders. The information was accessed via an online form (available at: https://forms.gle/L92wXsVaxxfPNgpE8). We evaluated through self-reported questionnaires in Portuguese:

• *Demographics and academics*: biological sex, age, course and academic shift.
• *Brazilian Portuguese version Pittsburgh Sleep Quality Index*: this is a questionnaire with 19 self-reported questions that assess sleep quality over a period of one month (16). The same has its version translated into Portuguese being validated and reliable, in addition it comes down to 7 components, subjective sleep quality, sleep latency, sleep duration, habitual sleep efficiency, sleep disturbances, sleep medication use and daytime dysfunction (4). Thus, each item is evaluated separately and according to the participant's response, the score can range from 0 to 3, calculating at the end of the 7 components up to 21 points. In this logic, the higher the score, the worse the quality of sleep. At a global level, with a score above 5 points, the participant already has severe impairments in at least 2 items or moderate impairments in 3 items (4).

### 2.6 Data collection

The study was conducted by a multidisciplinary work team, composed of researchers who are undergraduates and graduates from courses in the health area. Previously, the team participated in training on the study protocol (approach, application and retrieval of online questionnaires) and sanitary measures. During the training, the team also reviewed the online version of the consent form and the questionnaire. We conducted the survey in three steps. In the first step, we invited students to participate in the study in person, respecting all sanitary and institutional norms. At this stage, we explain the project and send the link with the free and informed consent form (via the social network, e.g., WhatsApp). In the second step, after electronic confirmation of signing the consent form, the participants answered the questionnaire (Q1, first application). In the third step, 2 weeks after the first stage, we resent the link, and the participants answered the same questionnaire again (Q2, second application). In the last two steps, we contacted the participants via WhatsApp only.

### 2.7 Statistical analysis

We performed all statistical analyzes in Stata software (version 15.0, Stata Corporation, College Station, TX, USA). We established the criterion of statistical significance of 95% (*p* ≤ 0.05) in the hypothesis tests. The normality of the variables was evaluated with the Shapiro-Wilk test. In the sensitivity analysis, we applied the chi-square goodness of fit test. In the reliability analysis, we applied Spearman's correlation coefficient, with a cutoff point ≥0.30 (for acceptable reliability) (17). In the validity analysis, we assessed the dimensionality using confirmatory factor analysis. We employed structural equation modeling (SEM) to address the previously hypothesized dimensionality (6, 8), following the assumptions for developing and validating questionnaires (7). We evaluated the global model adjustment using the fit indices: root mean square error of approximation (RMSEA < 0.08), standardized root mean square residual (SRMR < 0.08), Comparative Fit Index (CFI > 0.90), Tucker-Lewis Index (TLI > 0.90) (18).

## 3 Results

Table 1 shows the sensitivity analysis, sociodemographic and university conditions of the research sample. In the Q1 and Q2, the sample consisted of 195 and 117 students, respectively. The largest share in both Q1 and Q2 was female, with 68.7% and 72.7%, respectively. The age ranged from 18 to over 36 years old, with the highest number between 21 and 25 years old, with 44.6% in the first application and 45.7% in the second. The courses varied between the areas of health and also included law and administration. Regarding sensitivity, no significant differences were found (*p* > 0.05).

**Table 1:** Description of the sample and sensitivity analysis according to demographic and academic variables in the first and second application of the questionnaire.

Variables		Q1, %	Q2, %	*p* value
		*n* = 195	*n* = 117	
Biological sex	Male	31.3	27.4	0.36
	Female	68.7	72.7	
Age	Up to 20 years	23.6	26.7	0.63
	21–25 years	44.6	45.7	
	26–30 years	18.5	14.7	
	31–35 years	7.2	5.2	
	36 years or older	6.2	7.8	
Academic course	Nutrition	7.7	6.0	0.17
	Physical Education	24.0	24.8	
	Nursing	12.8	12.0	
	Aesthetics and Cosmetics	4.1	1.7	
	Physiotherapy	17.3	18.8	
	Law	9.7	11.1	
	Psychology	19.9	21.4	
	Social work	2.6	2.6	
	Administration	1.5	1.7	
Academic shift	Morning	20.1	20.5	0.92
	Evening	18.6	17.1	
	Night	61.4	62.4	

Q1, Questionnaire first application; Q2, Questionnaire second application. *P* value: Comparison between the distributions of the samples for the chi-square goodness-of-fit test.

**Figure 1:**
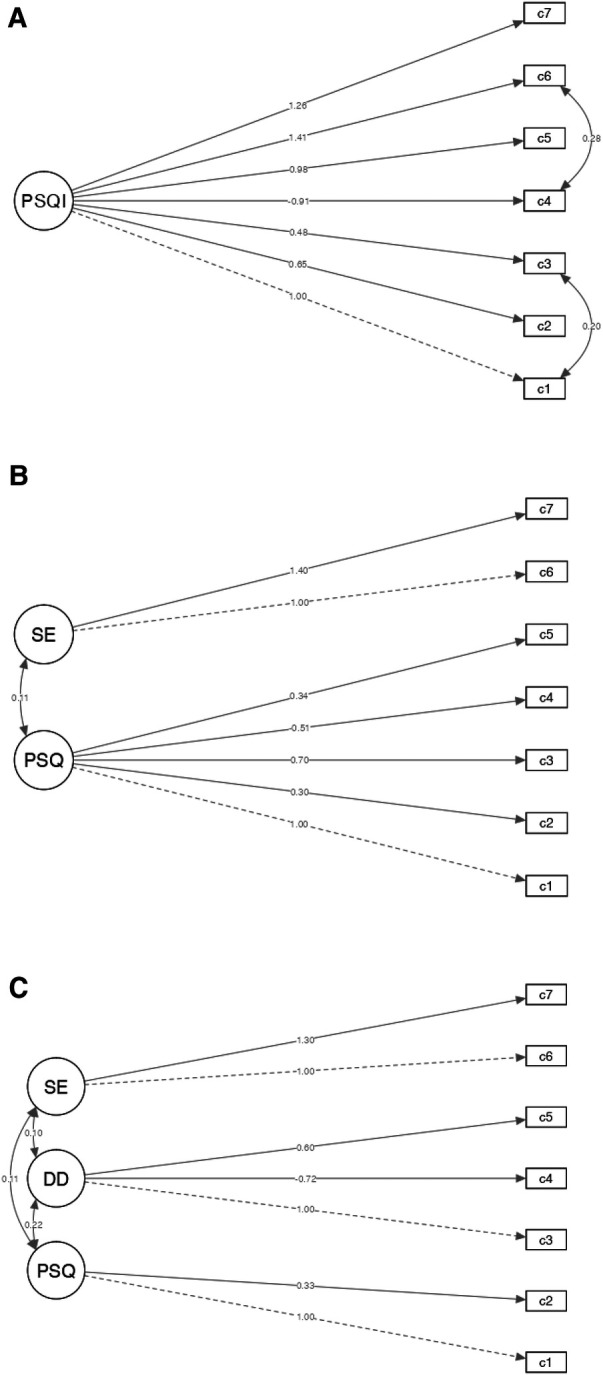
Coefficients for the one (**A**), two (**B**) and three-factor (**C**) model. DD, daily disturbance; SE; sleep efficiency; PSQ, perceived sleep quality; PSQI, Pittsburgh sleep quality index.

Table 2 shows the reliability (temporal stability) assessment of the PSQI-BR. In this sense, we observed the Spearman correlation coefficient ≥0.30, considering the reliability as acceptable. Table 3 shows the construct validity (confirmatory factor analysis) of the PSQI-BR. The dimensionalities tested (path diagrams) by SEM are available in the Figure 1. All fit indices for the one-factor model showed acceptable adjustment. We observed better adjustment for the one-factor (RMSEA = 0.019; SRMR = 0.041; CFI = 0.992; TLI = 0.986; Figure 1A) solution compared with two-factor (RMSEA = 0.099; SRMR = 0.070; CFI = 0.764; TLI = 0.619; Figure 1B) and three-factor (RMSEA = 0.108; SRMR = 0.066; CFI = 0.763; TLI = 0.548; Figure 1C) solutions, respectively.

**Table 2:** Descriptive results of Pittsburgh sleep quality Index (PSQI) scores and spearman correlation coefficient (rho) to estimate the reliability of the Pittsburgh sleep quality Index questionnaire in Brazilian adults.

PSQI components	Q1	Q2	rho*
	Median (p25–75)	Median (p25–75)	
Subjective Sleep Quality	1 (1.0–2.0)	1 (1.0–2.0)	0.49
Sleep Latency	2 (1.0–2.0)	2 (1.0–2.0)	0.46
Sleep Duration	1 (0.0–1.0)	1 (0.0–1.0)	0.59
Habitual Sleep Efficiency	3 (0.0–3.0)	1 (0.0–3.0)	0.50
Sleep Disturbances	2 (1.0–2.0)	1 (1.0–2.0)	0.53
Sleep Medication Use	0 (0.0–0.0)	0 (0.0–0.0)	0.63
Daytime Dysfunction	1 (1.0–2.0)	1 (1.0–2.0)	0.51
Total PSQI score	9 (7–10)	8 (6–10)	0.55

p25, percentile 25; p75, percentile 75, Q1, questionnaire first application; Q2, questionnaire second application.

*All presents significative correlation (*p* < 0.05).

**Table 3:** Confirmatory factor analysis of the of the Pittsburgh sleep quality Index questionnaire (PSQI) in Brazilian college students based on factor solution model.

Fit Indices	1-factor model	2-factor model	3-factor model
Chi-square (df)	12.7 (12)	35.4 (13)	33.4 (11)
RMSEA	0.019	0.099	0.108
SRMR	0.041	0.070	0.066
CFI	0.992	0.764	0.763
TLI	0.986	0.619	0.548

RMSEA, root mean square error of approximation (acceptable fit: <0.08); SRMR, standardized root mean square residual (acceptable fit: <0.08); CFI, comparative fit index (acceptable fit:  0.9); TLI, Tucker–Lewis index (acceptable fit: 0.9).

## 4 Discussion

The findings of our analysis suggest that online PSQI-BR questionnaire showed acceptable reliability and validity in college students from the low-income region. These results are very interesting for epidemiology, as they can help to identify the sleep quality in diverse and underrepresented populations in real-time data collection, even in conditions of restricted social contact, as experienced in a pandemic context.

Our study, conducted online among college students, revealed that a two-week interval offers acceptable temporal stability for the PSQI questionnaire. There is limited evidence on test-retest reliability in this population. As previously highlighted by Lash et al. (19), applying assessment methods in different populations and scenarios are essential steps to verify if the psychometric properties are maintained. However, the scientists identified acceptable temporal stability in Brazilian adolescents (9). This finding contributes to the ongoing debate regarding the reliability of subjective sleep quality reports. We offer potential explanations related to temporal stability. First, it is plausible that participants’ sleep patterns exhibit stability over a two-week period, contributing to the consistency observed in their responses. Additionally, the two-week interval may balance minimizing memory decay while capturing fluctuations in sleep patterns.

Our findings also indicated acceptable validity for the online version of the PSQI-BR in low-income college students. A comprehensive systematic review showed that the factor structure of PSQI was best explained by two-factor models (28.8% of the studies), followed by a one-factor model (20.0% of the studies) and three-factor models (17.8% of the studies) (6). Differently, our findings suggest the use of online PSQI-BR as a unitary construct in college students in contrast with studies that advocate the multifactorial application of this instrument (6, 8). We argue as a potential explanation that the observed construct of “sleep quality” covers a broad range of indicators relevant to sleep quality, as planned by PSQI developers (16). In the academic context, this is justified because longitudinal research during the pandemic shows that college students suffered from anxiety and poor sleep quality (20) mainly due to decreased social interactions (21), also they also developed “zoom fatigue” due to excessive hours of online classes (22). This is a reasonable explanation for why these constructs have presented higher loads in the analyzes we performed. For these reasons, and given the epidemiological evidence and usefulness backing up the unidimensional application of the PSQI in its ability to screen for sleep health, we advocate in favor of the global score of the PSQI as a potential marker of sleep quality in college students.

The present study is not without limitations, such as the sample selected by convenience, respondent bias (including social desirability) and the lack of epidemiological representativeness. Thus, the results should not be extended beyond the scope of the psychometric findings. Additionally, further studies should be conducted to confirm the validity of the PSQI in its online format across different population characteristics and backgrounds.

## 5 Conclusion

The online PSQI-BR has acceptable reliability and validity in Brazilian low-income regions. The questionnaire represents an easy and cost-effective way to measure remote sleep quality in college students from low-income regions.

## Data Availability

The raw data supporting the conclusions of this article will be made available by the authors, without undue reservation.
